# Study on Oxygen Supply Standard for Physical Health of Construction Personnel of High-Altitude Tunnels

**DOI:** 10.3390/ijerph13010064

**Published:** 2015-12-22

**Authors:** Chun Guo, Jianfeng Xu, Mingnian Wang, Tao Yan, Lu Yang, Zhitao Sun

**Affiliations:** 1 Key Laboratory of Transportation Tunnel Engineering, Ministry of Education, Southwest Jiaotong University, 111 Erhuan Rd, 610031 Chengdu, China; xujf@my.swjtu.edu.cn (J.X.); mingnianw@163.com (M.W.); swjtuyt@163.com (T.Y.); sunzhitaovip@163.com (Z.S.); 2 College of Foreign Languages, Southwest Jiaotong University, 111 Erhuan Rd, 610031 Chengdu, China; yanglu@swjtu.edu.cn

**Keywords:** high-altitude, oxygen supply, construction labor intensity, oxygen consumption, air density, equivalent PIO_2_

## Abstract

The low atmospheric pressure and low oxygen content in high-altitude environment have great impacts on the functions of human body. Especially for the personnel engaged in complicated physical labor such as tunnel construction, high altitude can cause a series of adverse physiological reactions, which may result in multiple high-altitude diseases and even death in severe cases. Artificial oxygen supply is required to ensure health and safety of construction personnel in hypoxic environments. However, there are no provisions for oxygen supply standard for tunnel construction personnel in high-altitude areas in current tunnel construction specifications. As a result, this paper has theoretically studied the impacts of high-altitude environment on human bodies, analyzed the relationship between labor intensity and oxygen consumption in high-altitude areas and determined the critical oxygen-supply altitude values for tunnel construction based on two different standard evaluation systems, *i.e.*, variation of air density and equivalent PIO_2_. In addition, it has finally determined the oxygen supply standard for construction personnel in high-altitude areas based on the relationship between construction labor intensity and oxygen consumption.

## 1. Introduction

With the rapid growth of China’s economy, the scale of road construction becomes larger year by year [1]. More and more high-altitude tunnel construction works have been placed on the agenda. According to incomplete statistics, for the moment, there are totally eight super long tunnels with the altitude above 3000 m having been completed or under construction, and there are more than 10 such tunnels under contemplation. The high-altitude tunnels currently completed and under construction in China, according to preliminary statistics, are listed in Table 1.

**Table 1:** Statistics of completed and under-construction high-altitude tunnels in China.

Number	Name of Tunnel	Length (m)	Portal Altitude (m)
1	Dabanshan Tunnel	1350	3792
2	Tielimaiti Tunnel	1894	3220
3	Yuximolegai Tunnel	1943	3200
4	Galongla Tunnel	3350	3250
5	Bai Mang Snow Mountain Tunnel	3951	4008
6	Zhegushan Tunnel	4448	3400
7	Queershan Tunnel (under construction)	7048	4300
8	Balangshan Tunnel (under construction)	7954	3852

According to related statistics, during construction of the tunnels of Qinghai–Tibet Railway during June–December 2001, as many as 36 cases of acute high altitude reaction syndrome [2] of construction personnel were reported. Among them, four cases happened at altitudes of 3000–4600 m, accounting for 11.1%; eight cases happened at altitudes of 4600–4800 m, accounting for 22.2%; and 24 cases happened at altitudes of 4800–5010 m, accounting for 66.7%.

Different from plain environment, high-altitude environment is characterized by low temperature, low atmospheric pressure and low content of oxygen. These differences have great physiological effects on the tunnel construction personnel, ranging from adverse physiological reactions [3] that may result in multiple high-altitude diseases to life risks in severe conditions. As a result, the safety of tunnel construction in high-altitude areas is significantly influenced. In particular, the low atmospheric pressure and oxygen content in high-altitude areas have enormous impacts on functions of human body. 

Air oxygen content refers to the amount of oxygen molecules contained in air per unit volume, which decreases with the increase of altitude. In addition, atmospheric pressure (PB) also greatly influences functions of human body, this is because oxygen partial pressure (PO_2_) [4] decreases with reduced PB while the water vapor pressure (PH_2_O) (47 mmHg) and CO_2_ pressure (PCO_2)_ (40 mmHg) in alveolus remain unchanged. If PB is 87 mmHg at the high altitude of 15,000 m, which is exactly equal to the sum of PH_2_O and PCO_2_ in alveolus, gas exchange cannot be conducted, even if pure O_2_ is supplied [5]. Therefore, the changes in PO_2_ and PB are important factors influencing human respiration and oxygen transfer in high-altitude areas. Refer to Table 2 for hypoxia responses [6] of human body at different altitudes. The equivalent oxygen concentration in Table 2 means that the mass of oxygen contained in altitude air per unit volume at local PB is same to the mass of certain concentration at the pressure of sea level.

**Table 2:** Hypoxia responses of human body at different altitudes.

Altitude (m)	0	1320	2400–3510	3510–6440	6440–10,860
PO_2_ (mmHg)	159	<137	121–106	106–76	76–46
Equivalent PB (atm)	1	0.86	0.76–0.67	0.67–0.48	<0.48
Hypoxia symptoms of human body [7]	Normal	Histocytes are in the hypoxia environment and slight symptoms are observed.	Deepened respiration, headache, quickened pulse and elevated blood pressure are observed. Coordinating functions of the body are degenerating and sleeping problems occur.	Fatigue, depression, attention deficit, dizziness and disorientation occur.	The symptoms of tinnitus, blurred vision, nausea and vomiting are observed. The body cannot move voluntarily. The patient can no longer speak and will become unconscious quickly.

It can be concluded that physical health of tunnel construction personnel in high altitude areas can be greatly impacted by air oxygen content. Therefore, artificial oxygen supply is of significant importance to ensure health and safety of construction personnel in hypoxic environment [8]. However, provisions for oxygen supply standard for tunnel construction personnel at altitude are dismissed. We hope our study will contribute to the working health and life security of high altitude workers, especially those are native plainsmen.

## 2. Related Diseases about Altitude Hypoxia and Human Adjustment to Altitude 

The amount of oxygen inhaled by tunnel construction personnel is directly influenced by oxygen concentration in the air. When air PO_2_ decreases, human respiration will be effected. The greater the oxygen concentration decreases, the more difficult respiration becomes. Human body will become hypoxic when oxygen concentration decreases to a certain degree, then working capacity will be reduced and various symptoms will occur.

Table 3 describes the relationship between hypoxia symptoms of construction personnel and air oxygen concentration [9]. 

**Table 3:** Relationship between acute hypoxia symptoms of construction personnel and oxygen concentration.

PO_2_ (mmHg)	Main Symptoms
129	No symptoms are observed when staying still. In movement, respiration frequency and resistance increase. Heart rate increases as well.
121	Respiration and heart rate increase. Dizziness and tinnitus occur. Judging capability is compromised and labor ability is lost.
76–91	Awareness and judgment become abnormal. Long-term exposure can be life threatening.
46–68	The symptoms of loss of consciousness and respiratory arrest occur. Heartbeat can only sustain for a short time and death will result without timely emergency treatment.

It is easy to see that severe lack of oxygen can imminently threaten the life of construction personnel. 

When workers enter high altitude, air pressure declines and air density decreases. The increase of pulmonary ventilation is an important physiologic compensation for low PO_2_ of inhaled gas (PIO_2_). As a result, constructors consume more volume of oxygen and output more CO_2_. Adaptive hyperventilation leads to a low PCO_2_ of artery blood. The blood tends to be alkaline and hypocarbia may occur. At the same time, the sensitivity of respiratory centers in brain to CO_2_ arises to decline the reaction threshold. This helps keep a relatively high level of pulmonary ventilation as the mechanism adjustment to hypoxia but may also result in respiratory alkalosis, which is not in favor of altitude acclimatization [10].

One typical disease caused by hypoxia is called acute mountain sickness (AMS), which refers to a series of acute hypoxia stress reaction of sea-level humans who have not established acclimatization of environmental adaptation mechanisms. The clinical manifestations of AMS are headache, hyperpnea, exertional dyspnea, cyanosis, anorexia, and abdominal distention [11]. 

Another disease named high-altitude pulmonary edema (HAPE) might be a greater threat to the health of construction workers. HAPE is a form of non-cardiogenic pulmonary edema that typically occurs in people who engage in vigorous physical activity without prior training. The attack of HAPE occurs quickly, does not cause fever, but does have premonitory symptoms such as headache, palpitation, insomnia, anorexia and nausea. The disease develops rapidly and requires active treatment. Otherwise, delay in therapy may result the death of patients [12].

What needs to be underlined is that the occurrence of HAPE at an altitude <2500 m is probably underestimated. André LG reported a series of 52 patients admitted for HAPE occurring at moderate altitude (1400 to 2400 m) over a nine-year period in a community hospital in the French Alps. They stated that HAPE patients are likely to be young, vacationing men, with no history of prior disease. Caution should be considered in the case of future high altitude travel [13]. 

High altitude cerebral edema (HACE) is usually thought to be the deuteropathy of AMS and HAPE. HACE is less common than AMS or HAPE but more mortal compared with the two diseases. It is known that the brain is the most oxygen-consuming organ of the human body when at rest. When people are exposed in low oxygen environment, human body will make adjustment measures to offset the influence of low PIO_2_. However, in the condition of urgent hypoxia, the brain blood circuit systems begin to have trouble and metabolic disorders happen. The patients of HACE firstly show disturbance of consciousness, then develop to deep coma, mental changes, clouding of consciousness and ataxia to some extent. Currently, the lowest recorded altitude of HACE attack is 2100 m, according to Dickinson. Without universal criteria for the definition and diagnoses, research on its epidemiology is also very limited. As a result, the actual morbidity of HACE is unclear [14]. 

Self *et al.* investigated possible physiological determinants of variability in hypoxia tolerance in subjects given a five-minute normobaric exposure to 7620 m equivalent. He reported that subjects with large total lung diffusion capacities for O_2_, those with the highest end-alveolar PO_2_ and the lowest mixed venous partial pressure (PvO_2_) at the end of the 5-min exposure, and those who maintained an O_2_ consumption rate that exceeded their resting levels, had the smallest declines in hemoglobin saturation [15]. 

A complex series of adaptive mechanisms follows the human being’s exposure to high altitude and the consequent hypobarism. However, the work of actual adaptive mechanisms depends on the rate of ascent and the altitude reached. If the mechanisms work, physiologic compensatory reaction will occur in human body and the acclimatization is successful. As to human beings who are exposed to high altitude, hypoxia stimulates the artery chemical sense receptors, which enhances ventilation through neuroregulation. Hyperventilation is necessary for high altitude adjustment. Hyperventilation replenishes more fresh inspiratory gas for pulmonary alveoli [16]. As a result, the PO_2_ at the interface between gas and blood increases and improve human ventilation.

In other words, the health and safety of construction workers faces severe threats from hypoxia. To protect human health at high altitudes and push the advance of altitude construction, oxygen supply is indispensable. However, at what altitude should oxygen supply start? We will introduce two different calculation methods to determine the specific altitudes that oxygen supply should start in the following section. 

## 3. Determination of Critical Oxygen-Supply Altitude for Construction Based on Changes in Air Density

Based on the influences of high-altitude environment on functions of human body, it can be seen that the oxidation capability is certain for some specific labor intensity of human body. As a result, the following assumptions have been made in this study:

(1) The amount of oxygen molecules required for the human body at set labor intensity is known.
(2) Volume concentration of oxygen does not change with the increase of altitude.
(3) Except altitude, all other factors are the same.

Considering that air densities and oxygen contents of low- and high-altitude areas are different and based on the above-mentioned assumptions, the amount of oxygen can be used as the basic value to obtain the maximum altitude that non-residents of high-altitude areas engaged in physical labor can adapt to i. The following equation can be established based on analyses: 

Sea-level oxygen density × limit oxygen concentration × unit volume = unit volume × standard oxygen concentration ×oxygen density at altitude

It is known that sea-level oxygen density is 1.429 kg/m^3^ and sea-level air density is 1.29 kg/m^3^. In low-altitude areas, the oxygen concentration possibly causing hypoxia of human body is 16%, while that possibly causing severe oxygen deficit is 12%. Theoretically, standard oxygen concentration is 21%. The relationships between altitude and air density, between density and pressure, and the above-mentioned equation are combined. Considering that densities of air and oxygen change in direct proportion, the critical oxygen-supply altitude that satisfies the requirements for both labor ability and life support of construction personnel can be determined. 

It is worth noting that heat emission during tunnel construction may increase the temperature in certain areas inside tunnel. According to Dalton’s Law of Partial Pressure, the air pressure in these areas will decline further due to the unchangeable volume in the tunnel. However, as to the input of fresh air and the heat exchange between air and rock in the tunnel, the temperature in long tunnels will cool down at places about 500 m away from construction face. The influence of temperature increase in long tunnels is very limited. However, the hypoxia situation in partial regions inside tunnel may be greater and therefore warrants caution and treatment to eliminate the passive effects on the working oxygen supply for workers.

### 3.1. Critical Oxygen-Supply Altitude Based on Labor Ability of Construction Personnel 

Taking account of the influences of changes in air density listed in Table 2, we know that if the equivalent oxygen concentration is not less than 16%, construction workers still maintain working ability. Naturally, we can take use of the relationship among gas density, altitude and gas pressure to calculate the critical altitude. The critical oxygen-supply altitude based on labor ability of construction personnel is determined as follows:

$$1.429\times 16\%\times 1=\rho_{oh}\times 21\%\times 1\Rightarrow \rho_{oh}=1.086kg/m^{3}$$

where 1.429 (unit: kg/m^3^) is sea-level oxygen density, 16% stands for the volume concentration of oxygen, 1 (unit: L) is the volume of oxygen, 21% is the volume fraction of oxygen, and ρ_oh_ (unit: kg/m^3^) is the oxygen density at certain altitude.

$$\frac{\rho_{oh}}{\rho_{osl}}=\frac{\rho_{ah}}{\rho_{asl}}\Rightarrow \rho_{ah}=\frac{\rho_{oh}\rho_{asl}}{\rho_{osl}}=\frac{1.806\times 1.29}{1.429}=0.982kg/m^{3}\text{ [17]}$$

where ρ_oh_ (unit: kg/m^3^) stands for the oxygen density at the altitude of h (unit: m), ρ_osl_ (unit: kg/m^3^) is the oxygen density at sea-level, ρ_ah_ (unit: kg/m^3^) means air density at the altitude of h (unit: m), and ρ_asl_ (unit: kg/m^3^) is air density at sea-level.

$$\frac{\rho_{a}}{\rho_{0}}=\frac{P_{a}}{P_{0}}\Rightarrow P_{a}=\frac{\rho_{a}P_{0}}{\rho_{0}}=\frac{0.982\times 101.325}{1.29}=76.96kPa\text{ [17]}$$

where P_a_ (unit: kPa) stands for the air pressure at the altitude of h (unit: m), P_o_ (unit: kPa) is the PO_2_ at sea-level, ρ_a_ (unit: kg/m^3^) means air density at the altitude of h, and ρ_o_ (unit: kg/m^3^) is oxygen density at sea-level (unit: m).

$$P_{a}=101.325\times {\left(1-\frac{h}{44329}\right)}^{5.255876}\Rightarrow h\approx 2500m\text{ [18]}$$

where h is the elevation height (unit: m) and P_a_ (unit: kPa) stands for the air partial pressure at the altitude of h (unit: m), while 101.325 (unit: kPa) is the air pressure at sea-level.

### 3.2. Critical Oxygen-Supply Altitude Based on Life Support of Construction Personnel

Similarly, the critical oxygen-supply altitude based on life support of construction personnel is:

$$1.429\times 12\%\times 1=\rho_{oh}\times 21\%\times 1\Rightarrow \rho_{oh}=0.817kg/m^{3}$$

where 1.429 (unit: kg/m^3^) is sea-level oxygen density, 12% stands for the volume concentration of oxygen, 1 (unit: L) is the volume of oxygen, 21% is the volume fraction of oxygen, and ρ_oh_ (unit: kg/m^3^) is the oxygen density at certain altitude.

$$\frac{\rho_{oh}}{\rho_{osl}}=\frac{\rho_{ah}}{\rho_{asl}}\Rightarrow \rho_{ah}=\frac{0.817\times 1.29}{1.429}=0.73kg/m^{3}$$

where ρ_oh_ (unit: kg/m^3^) stands for the oxygen density at the altitude of h(unit: m), ρ_osl_ (unit: kg/m^3^) is the oxygen density at sea-level, ρ_ah_ (unit: kg/m^3^) means air density at the altitude of h (unit: m), and ρ_asl_ (unit: kg/m^3^) is air density at sea-level.

$$\frac{\rho_{a}}{\rho_{0}}=\frac{P_{a}}{P_{0}}\Rightarrow P_{a}=\frac{\rho_{a}P_{0}}{\rho_{0}}=\frac{0.73\times 101.325}{1.29}=57.2kPa$$

where P_a_ (unit: Pa) stands for the air pressure at the altitude of h (unit: m), P_o_ (unit: kPa) is the PO_2_ at sea-level (unit: m), ρ_a_ (unit: kg/m^3^) means air density at the altitude of h, and ρ_o_ (unit: kg/m^3^) is oxygen density at sea-level (unit: m).

$$P_{a}=101.325\times {\left(1-\frac{h}{44329}\right)}^{5.255876}\Rightarrow h\approx 4600m$$

where h is the elevation height (unit: m), P_a_ (unit: kPa) stands for the air partial pressure at the altitude of h (unit: m) and 101.325 (unit: kPa) is the air pressure at sea-level.

By analyzing the relationship between two critical oxygen concentrations and corresponding altitudes, the possibilities that construction personnel will suffer hypoxia at altitudes >2500 m and severe hypoxia will occur when altitude exceeds 4600 m have been theoretically predicted. 

In Discussions on Labor Sanitation and Protection in High-altitude Areas [19], it is mentioned that when altitude is below 3000 m, the degree of blood oxygen saturation concentration of human body is usually above 90%. In this case, people will not develop hypoxia symptoms. Only slight increase of respiration frequency and heart rate is observed, which is called “the range with no obvious change”. At altitudes of 3000~4000 m, approximately one-third to one-fourth of people show inadaptability to high altitudes, when symptoms of quickened respiration and heart rate and hypoxia of organs occur. This range is called the “compensatory range”. At altitudes of 4500**~**6000 m, dysfunction of human body may occur, and this range is called the “dysfunction range”. At altitudes above 6000 m, hypoxia is aggravated, with the symptoms of collapse and coma. In this case, blood oxygen saturation concentration has decreased to below 70%. This range is usually called the “highly dangerous altitudes”. 

According to the above-mentioned statements and analyses on statistics of high-altitude syndromes of construction personnel working in high-altitude areas, the critical oxygen-supply altitude at which hypoxia may occur in the personnel is 2500 m, while the altitude at which severe hypoxia responses may occur is 4500 m.

## 4. Calculation Method for Critical Oxygen-Supply Altitude Based on Equivalent PIO_2_

The oxygen in the air is the only oxygen source for human body without auxiliary oxygen-supply measures.

PB and oxygen concentration are the direct factors for determining PO_2_. Their relationship can be expressed with the following equation: PO_2_ = PB × atmospheric oxygen concentration. In normal condition, volume concentration of oxygen in the air is 21%. Theoretically, it does not change along with altitudes. Therefore, PO_2_ mainly depends on atmospheric pressure. The higher the atmospheric pressure is, the higher the oxygen partial pressure will be. As the water vapor in the respiratory tract is fully saturated at normal body temperature and the PH_2_O in the trachea is 47 mmHg, PIO_2_ is lower than in the atmosphere. During each respiration, only some alveolar air is exchanged, while the oxygen in alveolar continues to disperse into alveolar capillaries [20,21,22]. Therefore, the oxygen partial pressure of alveolar air (PAO_2_) is about one-third lower than the PIO_2_ in the trachea. The oxygen contained in alveolar air can be dispersed into the blood. In addition, PAO_2_ is also related to alveolar ventilation and oxygen consumption of the body. When alveolar ventilation decreases or oxygen consumption of the body increases, PAO_2_ is reduced and *vice versa*.

PAO_2_ = (PB − PH_2_O) × FiO_2_ − 1.25 × PCO_2_.

FiO_2_ stands for the fraction of inspired oxygen.

According to proceeding equation, we can calculate the PAO_2_ at different altitudes and the results is shown in Table 4.

**Table 4:** Changes in atmospheric pressure, atmospheric oxygen partial pressure, oxygen partial pressure of inhaled gas and alveolar oxygen partial pressure at different altitudes.

Altitude (m)	PB (mmHg)	PO_2_ (mmHg)	PIO_2_ (mmHg)	PAO_2n_(mmHg)
0	760	159	149	105
1000	674	141	131	90
2000	596	125	115	70
3000	530	110	100	62
4000	460	98	87	50
5000	405	85	74	45
6000	355	74	64	40
7000	310	65	55	35
8000	270	56	47	30

PIO_2_ is a main factor for oxygen to influence human body. Decreased oxygen concentration and PB will result in decreased PO_2_ and thus lead to hypoxia of human body. In high-altitude areas, equivalent oxygen concentration is relatively low and PIO_2_ decreases with atmospheric PO_2_. To determine the oxygen deficit standard more conveniently, it is more intuitive to measure the degree of oxygen deficit with altitude instead of oxygen concentration in high-altitude areas. Therefore, oxygen concentration can be converted into altitude to indicate content of oxygen. 

Calculation formula for PB at the altitude below 11,000 m is shown as Equation (1):

(1) $$PB=101.325\times {\left(1-\frac{h}{44329}\right)}^{5.255876}$$

The unit of PB in Equation (1) is kPa and h stands for altitude height (unit: m).

The common calculation formula for PIO_2_ is shown as Equation (2):

(2) $$PIO_{2}=FiO_{2}(PB-PH_{2}O)$$

The unit of PIO_2_ in Equation (2) is kPa and the value of PH_2_O is 6.27 kPa. 

By combining the above equations, it is concluded that the air with a given oxygen concentration at a specified altitude can be related to the air with a normal oxygen concentration (21%) at a specified altitude. Refer to Equation (3), where the altitude is h_1_, oxygen concentration is FiO_2_ and equivalent altitude is h_2_:

(3) $$h_{2}=a\times \left\{1-\sqrt[{b}]{\frac{FiO_{2}\left[101.325{\left(1-h_{1}/a\right)}^{b}-6.27\right]}{21.217455}}\right\}$$

where, a = 44,329 and b = 5.255876, a and b are both constants.

## 5. Determination of Critical Oxygen-Supply Altitude for Construction Based on Equivalent PIO_2_

Considering the impact of equivalent PIO_2_, Equation (3) is adopted for calculation, and the critical oxygen-supply altitude for construction personnel is obtained as follows [23]:

### 5.1. Critical Oxygen-Supply Altitude Based on Labor Ability of Personnel

Critical oxygen-supply altitude based on labor ability of personnel:

$$\begin{array}{l} h_{2}=a\times \left\{1-\sqrt[{b}]{\frac{FiO_{2}\left[101.325{\left(1-h_{1}/a\right)}^{b}-6.27\right]}{21.217455}}\right\}\\ =44329\times \left\{1-\sqrt[{5.255876}]{\frac{0.16\times \left[101.325{\left(1-0/44329\right)}^{5.255876}-6.27\right]}{21.217455}}\right\}\\ \approx 2700m \end{array}$$

### 5.2. Critical Oxygen-Supply Altitude Based on Life Support of Personnel

Critical oxygen-supply altitude based on life support of personnel:

$$\begin{array}{l} h_{2}=a\times \left\{1-\sqrt[{b}]{\frac{FiO_{2}\left[101.325{\left(1-h_{1}/a\right)}^{b}-6.27\right]}{21.217455}}\right\}\\ =44329\times \left\{1-\sqrt[{5.255876}]{\frac{0.12\times \left[101.325{\left(1-0/44329\right)}^{5.255876}-6.27\right]}{21.217455}}\right\}\\ \approx 4950m \end{array}$$

Analyses and calculations are conducted on two critical oxygen concentrations based on the principle of equivalent PIO_2_. In theory, the possibilities that construction personnel will suffer hypoxia at altitudes >2700 m and severe hypoxia will occur when altitude exceeds 4950 m have been theoretically predicted. For the purpose of guaranteeing personal safety, the altitude for oxygen deficit is determined rather conservatively, *i.e.*, the critical oxygen-supply altitude for possible hypoxia responses is 2500 m, while the critical oxygen-supply altitude for severe hypoxia responses is 4500 m. 

Based on calculations of above-mentioned critical oxygen-supply altitudes, it is finally decided that the critical altitude at which oxygen must be supplied to construction personnel is 4500 m, while that at which labor ability of construction personnel shall be considered is 2500 m.

## 6. Oxygen Supply for Construction at Different Altitudes

Although the starting oxygen supply heights are decided in previous sections, the oxygen supply criteria for different altitudes are undetermined. By supplying oxygen, the PIO_2_ at different altitudes can reach the level of low-altitude areas. The following is the equivalent oxygen supply theory to calculate the needed oxygen in per unit volume of air at altitude:

PIO_2_ = (PB − PH_2_O) × FiO_2_ (4)

According to Equation (4) and the values of PB in Table 4, the oxygen supply for increasing oxygen contents at higher altitudes to the level of sea level can be calculated [24], as shown in Table 5. FiO_2_ increases with the rise of altitudes. 

**Table 5:** FIO_2_ required for maintaining sea-level PO_2_.

Altitude (m)	1000	2000	3000	4000	5000	6000	7000
FiO_2_ (%)	23.76	27.14	30.85	36.08	41.32	48.38	56.65

Compared with the FiO_2_ of sea level (21%), the increase of FiO_2_ with altitude increase is more and more obvious. For example, the FiO_2_ difference between sea level and the altitude of 1000 m is only 2.76 percentage points, but the difference increase to 7.08 percentage points between the altitude of 5000 m and 6000 m. The reason is the accelerating increase of air density difference at higher altitude. 

## 7. Relationship between Labor Intensity in Tunnel Construction and Oxygen Consumption in High-Altitude Areas

The intensity of short-interval labor is usually greater than that of long-interval labor. If intensity of individual labor were classified as different labor intensity grades based on energy consumption, the grade values would mean the rank in intensity classification would be higher than expected. Therefore, this method is seldom adopted presently. To further determine the relationship between types of work and oxygen demand and consumption as well as determine the oxygen-supply plan for tunnel construction personnel in high-altitude areas, oxygen consumption is thus taken as the criterion for classifying labor grade [25,26].

Based on classification on intensity of physical work of China [27] and the oxygen consumption and amount of inspired air for each grade of labor intensity, the volumes of oxygen needed by human body at the PB of different altitudes are estimated and shown in Table 6. The grades of labor intensity of several main procedures in tunnel construction and the oxygen consumption of sea level are determined, as shown in Table 7.

**Table 6:** Minimum oxygen consumption under different labor intensities at different altitudes (L/min).

Labor Intensity	0 m	1000 m	2000 m	3000 m	4000 m	5000 m
Very light	<0.5	<0.6	<0.6	<0.7	<0.8	<0.9
Light	0.5	0.6	0.6	0.7	0.8	0.9
Moderate	1	1.1	1.3	1.4	1.6	1.8
Heavy	1.5	1.7	1.9	2.2	2.4	2.7
Very heavy	2	2.3	2.5	2.9	3.2	3.6
Extremely heavy	2.5	2.8	3.2	3.6	4	4.5

**Table 7:** Grades of labor intensity of main procedures in tunnel construction and oxygen consumption at sea level.

Procedure	Grade of Labor Intensity	Oxygen Consumption (L/min)
Drilling and blasting	Very heavy labor	2.0–2.5
Shotcrete	Heavy labor	1.5–2.0
Formwork lining	Heavy labor	1.5–2.0
Paving waterproof board	Moderate labor	1.0–1.5
Slag loading	Moderate labor	1.0–1.5
Slag tapping	Light labor	0.5–1.0

Air volume expands and density decreases at an elevated altitude, which results in decrease in oxygen content in the air. As the amount of oxygen needed by human body is constant, the volumetric oxygen consumption of human body at local altitude can be obtained via mass conversion. 

Take the Balangshan Tunnel under construction as an example: The altitude of the tunnel is about 4000 m, where air density is 0.802 kg/m^3^, approximately 62% of the air density at sea level. As oxygen varies in direct proportion with air, oxygen content in the air is also 62% of that at sea level. New grades of labor intensity and oxygen consumption [28,29] are calculated at local PB via mass transformation, as shown in Table 8. 

**Table 8:** Grade of labor intensity and oxygen consumption at the altitude of 4000 m.

Procedure	Grade of Labor Intensity	Oxygen Consumption (L/min)
Drilling and blasting	Extremely heavy labor	3.2–4
Shotcrete	Extremely heavy labor	2.4–3.2
Formwork lining	Extremely heavy labor	2.4–3.2
Paving waterproof board	Very heavy labor	1.6–2.4
Slag loading	Very heavy labor	1.6–2.4
Slag tapping	Heavy labor	0.8–1.6

By analyzing the relationship between the grades of labor intensity of main procedures in tunnel construction and oxygen consumption at sea level, it can be seen that labor intensity and oxygen consumption increase in a gradual but significant manner at elevated altitudes.

## 8. Determination of Oxygen-Supply Standard for Tunnel Construction Personnel in High-Altitude Areas

It is usually believed that the volumetric percentage of oxygen in the air is constant in both high- and low-altitude areas. However, air density and oxygen density decrease at higher altitudes, resulting in decrease in oxygen content of the same volume. When people are engaged in manual work, the amount of oxygen needed for energy consumption will be constant if labor intensity is unchanged. In *Ventilation and Oxygen Consumption Rate* [30], it is believed that oxygen consumption of human body is 4.5 mL per 100 mL of ventilation. On this basis, the ventilation for different grades of labor intensity of human body at different altitudes can be estimated, as shown in Table 9.

**Table 9:** Minimum ventilation of human body under different labor intensities at different altitudes (L/min).

Labor Intensity	0 m	1000 m	2000 m	3000 m	4000 m	5000 m
Very light	<11.1	<12.5	<14.2	<16.1	<17.9	<20.0
Light	11.1	12.5	14.2	16.1	17.9	20.0
Moderate	22.2	25.1	28.3	32.2	35.8	39.9
Heavy	33.3	37.6	42.5	48.3	53.7	59.9
Very heavy	44.4	50.1	56.6	64.4	71.6	79.9
Extremely heavy	55.6	62.6	70.8	80.5	89.5	99.8

Usually, ventilation of a human body not engaged in work is about 10 L/min, and maximum ventilation range is 70~120 L/min. To protect laborers’ health, the conservative value of 70 L/min is adopted as the maximum ventilation for work [31]. For the purpose of improving working efficiency and based on the ventilation values given in the table, it is suggested that extremely heavy labor not be conducted at the altitude of 3000 m or above and very heavy labor not be conducted at the altitude of 4000 m or above, at which moderate or lighter labor is recommended.

On the basis of relevant studies, it has been decided that the oxygen-supply altitude for guaranteeing labor ability of construction personnel is 2500 m. By considering classification of labor intensity and minimum oxygen consumption under different labor intensities at different altitudes given in Table 6, the oxygen-supply standard based on labor ability of personnel can be determined, as shown in Table 10.

**Table 10:** Oxygen-supply standard based on labor ability of construction personnel.

Labor Intensity	Oxygen Supply (L/min)							
	2500 m	3000 m	3500 m	4000 m	4500 m	5000 m	5500 m	6000 m
Very light	0	0.04	0.09	0.14	0.20	0.26	0.32	0.40
Light	0	0.09	0.18	0.29	0.40	0.52	0.65	0.79
Moderate	0	0.13	0.28	0.43	0.60	0.78	0.97	1.19
Heavy	0	0.18	0.37	0.57	0.80	1.04	1.30	1.58
Very heavy	0	0.22	0.46	0.72	1.00	1.30	1.62	1.98
Extremely heavy	0	0.27	0.55	0.86	1.19	1.56	1.95	2.37

Based on relevant studies, it has been decided that the oxygen-supply altitude for guaranteeing safety of construction personnel is 4500 m. By considering classification of labor intensity and minimum oxygen consumption under different labor intensities at different altitudes given in Table 6, the oxygen-supply standard based on safety of personnel can be determined, as shown in Table 11.

**Table 11:** Oxygen-supply standard based on safety of construction personnel.

Labor Intensity	Oxygen Supply (L/min)			
	4500 m	5000 m	5500 m	6000 m
Very light	0	0.06	0.13	0.20
Light	0	0.12	0.25	0.39
Moderate	0	0.18	0.38	0.59
Heavy	0	0.24	0.50	0.78
Very heavy	0	0.30	0.63	0.98
Extremely heavy	0	0.36	0.75	1.18

## 9. Conclusions

(1) Two standards, respectively, on the bases of labor ability and life support of construction personnel in high-altitude areas have been brought forward based on mass conservation of oxygen at sea level and in high-altitude areas, according to the impact of oxygen concentration on functions of human body during labor at sea level. An equation combining the oxygen mass conservation equation and the relationship between altitude and PIO_2_ of human body and oxygen concentration has been established (see Equation (3)). 

(2) The altitudes for oxygen supply based on labor ability and life support are separately calculated with two methods. It is finally determined that the critical altitude at which oxygen must be supplied to construction personnel is 4500 m, while that at which labor ability of construction personnel should be considered is 2500 m.

(3) The joint impact of labor intensity and altitude on oxygen consumption of human body has been proposed for the first time, and the oxygen consumptions of construction personnel under different labor intensities are shown in Table 6. The standard for oxygen supply based on labor ability is proposed, as shown in Table 10. The standard for oxygen supply based on safety is proposed, as shown in Table 11. 

## References

[1] Deng Y. (2009). Discussion of development trend of long tunnel construction in China. Railway Constr. Technol..

[2] Sun Y.F., Zen F.L. (2005). Research on the occupational hazards and their control measures of Qinghai-Tibet Railway constructors. Chin. J. Ind. Hyg. Occ. Dis..

[3] Bian S.Z., Jin J., Li Q.N., Yu J., Tang C.F., Rao R.S., Yu S.Y., Zhao X.H., Qin J., Huang L. (2015). Hemodynamic characteristics of high-altitude headache following acute high altitude exposure at 3700 m in young Chinese men. J. Headache Pain.

[4] Dings J., Meixensberger J., Jäger A., Roosen K. (1998). Clinical experience with 118 brain tissue oxygen partial pressure catheter probes. Neurosurgery.

[5] Lv Y.D. (1995). High altitude medicine and physiology. Science and Technology.

[6] Kawashima A., Kubo K., Kobayashi T., Sekiguchi M. (1989). Hemodynamic responses to acute hypoxia, hypobaria and exercise in subjects susceptible to high-altitude pulmonary edema. J. Appl. Phys..

[7] Lawley J.S., Alperin N., Bagci A.M., Lee S.H., Mullins P.G., Oliver S.J., Macdonald J.H. (2014). Normobaric hypoxia and symptoms of acute mountain sickness: Elevated brain volume and intracranial hypertension. Ann. Neurol..

[8] China Traffic Construction Co., LTD. (2015). Safety Technical Specifications for Highway Engineering Construction.

[9] Xiao X.Z. (2004). Pathophysiology.

[10] Moore L.G., Niermeyer S., Zamudio S. (1998). Human adaptation to high altitude: Regional and life-cycle perspectives. Am. J. Phys. Anthropol..

[11] Zhang X.Z. (2009). Acute high altitude disease. People’s Milit. Surg..

[12] Zhang L.J. (2013). High-altitude pulmonary edema. J. Med. Radiol. Technol..

[13] André L.G., Xavier L., Monique M., Claude M. (2003). High-altitude pulmonary edema at moderate altitude (<2400 m; 7870 feet)—A series of 52 patients. Chest.

[14] Hackett P.H., Roach R.C. (2005). High-altitude cerebral edema. J. Qinghai Med. Coll..

[15] Self D.A., Mandella J.G., White V.L., Burian D. (2013). Physiological determinants of human acute hypoxia Tolerance. Hypoxia.

[16] Sanborn M.R., Edsell M.E., Kim M.N. (2015). Cerebral hemodynamics at altitude effects of hyperventilation and acclimatization on cerebral blood flow and oxygenation. Wild. Environ. Med..

[17] Shi F.K. (1991). Narrate Dalton law vividly with TP transparency. Chin. J. Chem. Edu..

[18] Fang X.B. (2005). The discussion about the relationship between air pressure and altitude height once more. J. Ankang Univ..

[19] Yu Y.Z., Li T.L., Liu Z.L. (1978). Discussions on labor sanitation and protection in high-altitude areas. Health Res..

[20] Hechtman H.B., Reid M.H., Dorn B.C., Weisel R.D. (1973). Indicator dilution measurements of lung volumes and alveolar air exchange during breathing. J. Clin. Investig..

[21] Gilbert R., Auchincloss J.H., Kuppinger M., Thomas M.V. (1979). Stability of the arterial/alveolar oxygen partial pressure ratio. Effects of low ventilation/perfusion regions. Crit. Care Med..

[22] Borland C.D., Cox Y. (1991). Effect of varying alveolar oxygen partial pressure on diffusing capacity for nitric oxide and carbon monoxide, membrane diffusing capacity and lung capillary blood volume. Clin. Sci..

[23] Balykin M.V., Karkobatov K.D. (2012). Systemic and organ mechanisms of the organism oxygen supply in high altitude. Ross Fiziol Zh Im IM Sechenova..

[24] Liu Y.S., Cui H.S., Liu W.H., Feng K.X., Le K., Yu Q.X. (2005). Research on oxygen supply technology of tunnel construction in high-altitude regions. Mining Metall..

[25] Fang R., Yu S.H., Xia C.C., Ci D., Li J.L. (2014). Research on key oxygen-supply technology of extra-long tunnel construction in high-altitude and cold regions. Railway Transp. Technol..

[26] Yan T., Wang M.N., Guo C., Chen H.B., Xie W.Q. (2015). Research on key technology of diffusing oxygen-supply of high-altitude extra-long railway tunnel. Modern Tunnel Technol..

[27] National Bureau of Standards of China Classification on intensity of physical work. http://baike.baidu.com/link?url=1hxkNtaCdFyqyi_SI219oBPOh9EnYu2JXfXHC7irWlE-EoL-aF5knHgzut4UW_CR_MqUrvMLdegrEk-twARWQa.

[28] Yang L., Wang X., Ma M. (1995). Analysis on the service condition of Chinese classification on intensity of physical work—Comment on physical work load. Ind. Health Occ. Diseases.

[29] Zhang S.J., Li J.G., Song C.P. (1994). Research on the Chinese classification on intensity of physical work on plateau. J. Chin. Ind. Hyg. Occ. Diseases..

[30] Yu Y.Z., Li T.L. (1980). Ventilation and oxygen consumption rate. Health Res..

[31] Yang X., Liu Y.S., Shen M., Liu W.H., Zhang H., Li Y.L., Meng Y. (2009). Maximum safe concentration of oxygen-enriched atmosphere in high altitude. J. Univ. Sci. Technol..

